# Porous Co_2_VO_4_ Nanodisk as a High-Energy and Fast-Charging Anode for Lithium-Ion Batteries

**DOI:** 10.1007/s40820-021-00758-5

**Published:** 2021-12-02

**Authors:** Jinghui Ren, Zhenyu Wang, Peng Xu, Cong Wang, Fei Gao, Decheng Zhao, Shupei Liu, Han Yang, Di Wang, Chunming Niu, Yusong Zhu, Yutong Wu, Xiang Liu, Zhoulu Wang, Yi Zhang

**Affiliations:** 1 School of Energy Science and Engineering, Nanjing Tech University, Nanjing, 211816 People’s Republic of China; 2 Center of Nanomaterials for Renewable Energy, State Key Laboratory of Electrical Insulation and Power Equipment, School of Electrical Engineering, Xi’an Jiaotong University, Xi’an, 710054 People’s Republic of China; 3 Department of Computational Materials Design, Max-Planck-Insitut Für Eisenforschung GmbH, Max-Planck-Straße 1, 40237 Düsseldorf, Germany

**Keywords:** Lithium-ion batteries, Anode, Fast-charging, High-energy, Cobalt vanadate oxide

## Abstract

High-energy–density lithium-ion batteries (LIBs) that can be safely fast-charged are desirable for electric vehicles. However, sub-optimal lithiation potential and low capacity of commonly used LIBs anode cause safety issues and low energy density. Here we hypothesize that a cobalt vanadate oxide, Co_2_VO_4_, can be attractive anode material for fast-charging LIBs due to its high capacity (~ 1000 mAh g^−1^) and safe lithiation potential (~ 0.65 V vs. Li^+^/Li). The Li^+^ diffusion coefficient of Co_2_VO_4_ is evaluated by theoretical calculation to be as high as 3.15 × 10^–10^ cm^2^ s^−1^, proving Co_2_VO_4_ a promising anode in fast-charging LIBs. A hexagonal porous Co_2_VO_4_ nanodisk (PCVO ND) structure is designed accordingly, featuring a high specific surface area of 74.57 m^2^ g^−1^ and numerous pores with a pore size of 14 nm. This unique structure succeeds in enhancing Li^+^ and electron transfer, leading to superior fast-charging performance than current commercial anodes. As a result, the PCVO ND shows a high initial reversible capacity of 911.0 mAh g^−1^ at 0.4 C, excellent fast-charging capacity (344.3 mAh g^−1^ at 10 C for 1000 cycles), outstanding long-term cycling stability (only 0.024% capacity loss per cycle at 10 C for 1000 cycles), confirming the commercial feasibility of PCVO ND in fast-charging LIBs.

**Supplementary Information:**

The online version contains supplementary material available at 10.1007/s40820-021-00758-5.

## Highlights

- The Li^+^ diffusion coefficient of Co_2_VO_4_ is evaluated by theoretical calculation to be as high as 3.15 × 10^–10^ cm^2^ s^−1^, theoretically proving Co_2_VO_4_ a promising anode in fast-charging lithium-ion batteries.
- A hexagonal porous Co_2_VO_4_ nanodisk (PCVO ND) structure is designed, featuring a high specific surface area of 74.57 m^2^ g^−1^ and numerous pores with a pore size of 14 nm.
- The PCVO ND shows excellent fast-charging performance (a high average capacity of 344.3 mAh g^−1^ at 10 C for 1000 cycles with only 0.024% capacity loss per cycle for 1000 cycles).

**Supplementary Information:**

The online version contains supplementary material available at 10.1007/s40820-021-00758-5.

## Graphical abstract

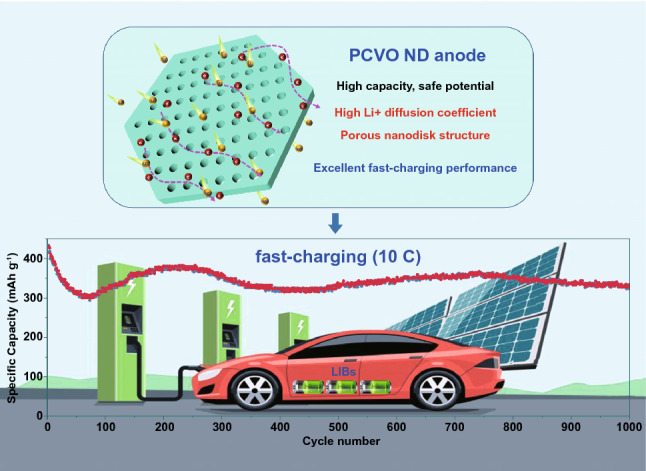

## Introduction

Lithium-ion batteries (LIBs) are widely used for portable electronic devices in the past decade because of their high-energy density, long cycling life, low self-discharge, the absence of memory effect, and low environmental impact [1, 2]. In recent years, LIBs have aroused extensive attention as the power source for electric vehicles (EVs). At present, EVs have advanced rapidly in terms of both range and cost yet there is still a lack of consumer acceptance and low market penetration of current EVs [3]. The main reason is that the current LIBs in EVs require a long charging time (hours or longer) in a safe manner compare with conventional gasoline vehicles [4]. Thus, improving the fast-charging performance of LIBs is critical to mainstream adoption of EVs for a sustainable future.

As a result, the US Department of Energy has set a goal for designing a LIBs pack that can withstand a 200-mile charge in only 7.5 min [5]. Achieving this goal requires LIBs' anode materials can be charged to a specific capacity of 175 to 200 mAh g^−1^ at a current density of > 10 A g^−1^ [6]. Nevertheless, current LIBs commonly used anodes do not meet the requirements. Graphite is the most popular anode material for commercial LIBs because of its high theoretical capacity (372 mAh g^−1^), high cycling stability, and low cost [7, 8]. However, graphite is unsatisfactory in fast-charging LIBs due to its lithiation potential (~ 0.1 V vs. Li^+^/Li), which is close to lithium metal's potential (0 V vs. Li^+^/Li) that lithium dendrites can quickly grow while charging at high rates. The lithium dendrites on the surface of the anode can puncture the polymer separator and connect the positive and negative electrodes, causing a short circuit that will lead to fire or explosion [9].

As an alternative, spinel Li_4_Ti_5_O_12_, with a high working potential (~ 1.55 V vs. Li^+^/Li), can be fast-charged without the concern of lithium dendrites growth and safety issues [10, 11]. Therefore, Li_4_Ti_5_O_12_ has been widely studied and already used as an anode for fast-charging LIBs in electric buses. Nevertheless, the energy density of LIBs based on Li_4_Ti_5_O_12_ anode is severely limited by the low theoretical capacity of Li_4_Ti_5_O_12_ (175 mAh g^−1^) and high lithiation potential [12]. Besides graphite and Li_4_Ti_5_O_12_, Si is also a commercially available LIBs anode. Si possesses an ultrahigh theoretical capacity (4200 mAh g^−1^) and a low, yet safe lithiation potential (~ 0.22 V vs. Li^+^/Li), holding great promise in safe and high-energy anodes for LIBs [13, 14]. However, its fast-charging performance deteriorates because of the enormous volume change (~ 400%) and low Li^+^ diffusion coefficient (4.60 × 10^–14^ cm^2^ s^−1^) during cycling [15]. Thus, it is essential to develop novel anode materials for fast-charging LIBs simultaneously featuring suitable lithiation potential, high capacity, and fast lithium diffusion.

Recently a novel spinel cobalt vanadate oxide (Co_2_VO_4_) exhibits several attractive features in LIBs, such as suitable Li^+^ lithiation potential, high capacity, and high electrochemical stability [16]. Through mixed intercalation and conversion reaction mechanism originated from the multiple valence states of V and Co element, the Co_2_VO_4_ anode can deliver a high stable capacity of 706.8 mAh g^−1^ at 1.0 A g^−1^ after 1000 cycles with a relatively low, yet safe Li^+^ lithiation potential (~ 0.65 V vs. Li^+^/Li) [17]. Based on these good electrochemical performances, we hypothesize that Co_2_VO_4_ can be an attractive anode material for fast-charging LIBs. We first evaluate the Li^+^ diffusion coefficient of Co_2_VO_4_ by ab initio molecular dynamics (AIMD) calculation which shows the Li^+^ diffusion coefficient of Co_2_VO_4_ is as high as 3.15 × 10^–10^ cm^2^ s^−1^, proving Co_2_VO_4_ a promising anode in fast-charging LIBs. Then we design a hexagonal porous nanodisk (PCVO ND) structure which can reduce the electron and ion diffusion length, further enhancing the lithium ions and electrons transfer. Moreover, numerous internal pores of PCVO ND can buffer the volume change of PCVO ND, leading to good long-term cycling stability at a high rate (maintain a high stable capacity of 344.3 mAh g^−1^ at 10 C after 1000 cycles with only 0.024% capacity loss per cycle). The result demonstrates that PCVO ND is an ideal high-energy fast-charging anode for LIBs.

## Materials and Methods

### Theoretical Calculation Details

Our calculations were performed using the plane-wave Vienna Ab initio Simulation Package (VASP) [18–20] within a projector augmented-wave (PAW) pseudo-potential method [21]. The Perdew-Burke-Ernzerhof (PBE) [22] form of GGA exchange–correlation functional was applied. The Hubbard U correction [23] is introduced to describe the effect of localized *d* electrons of Co and V ions. The U value of Co ions is 5.0 eV, while the U value of V ions is 2.7 eV. To study the Li diffusion, ab initio molecular dynamics (AIMD) simulation was carried out under the Born–Oppenheimer approximation. To speed up diffusion and shorten the simulation time scale, AIMD simulations were performed at 500, 750, 1000, 1250, and 1500 K with a time step of 2 fs after reaching the desired temperature. All these structures were equilibrated for 50 ps before diffusion properties were analyzed. MD simulations in the NVT ensemble with a Nosé-Hoover thermostat [24] were applied. The supercell of Co_2_VO_4_ used for diffusion study comprises 2 × 2 × 2 primitive unit cells (16 formula units). Li^+^ are intercalated in the interstitial sites between CoO_6_ octahedra and VO_4_ tetrahedra, where each of Li^+^ is surrounded by 6 O ions. 3Li^+^ are intercalated in the 2 formula units of Co_2_VO_4_. A kinetic energy cutoff of 500 eV and a Γ-centered 1 × 1 × 1 k-point mesh was adopted to carry out sampling integral for Brillouin zone. These parameters are necessary for convergence of the total energy to within 10^–5^ eV per atom and force less than 0.01 Ev Å^−1^ per atom.

The diffusion coefficient (*D*) is defined as the slope of the average mean square displacement (MSD) over 2*dt*:

1 $$D = \mathop {lim}\limits_{x \to \infty } \frac{1}{2dt}\overline{{[\vec{r}\left( t \right)]^{2} }}$$

where $\vec{r}\left( t \right)$ is the displacement of the *i*-th lithium ion at time *t*.

The average mean square displacement (MSD) is defined as a measure of the deviation of the position of a particle with respect to a reference position over time:

2 $$\overline{{[\vec{r}\left( t \right)]^{2} }} = \frac{1}{N}\mathop \sum \limits_{N}^{i = 1} \overline{{\left[ {\vec{r}\left( {t + t_{0} } \right)]^{2} - } \right[\vec{r}\left( {t_{0} } \right)]^{2} }}$$

where *N* is the total number of Li^+^ in the system.

### Materials Synthesis

**Fig. 1:**
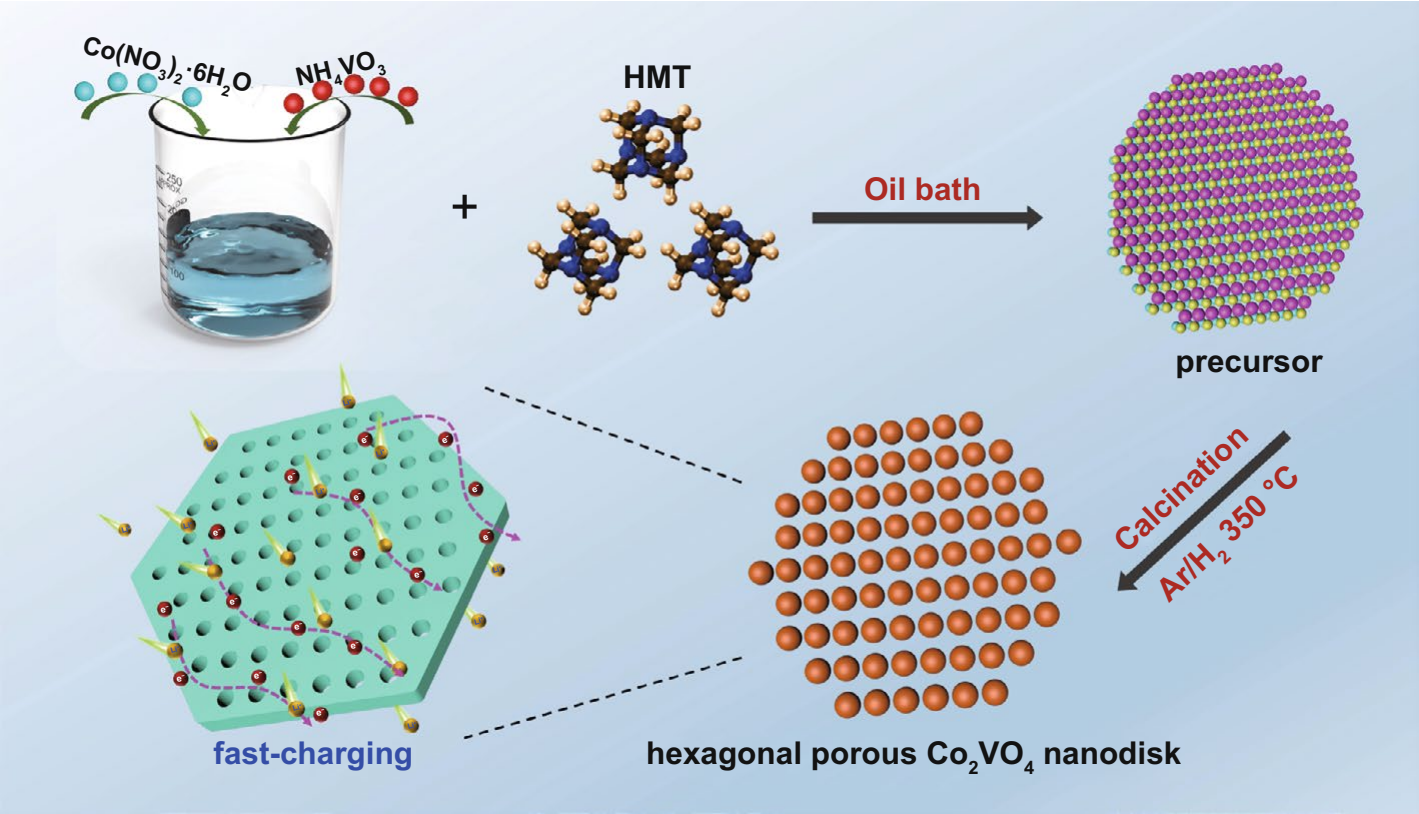
Schematic illustration of PCVO ND synthesis

The hexagonal porous Co_2_VO_4_ nanodisk (PCVO ND) was synthesized by a simple, low cost and scalable route. First, 0.94 g NH_4_VO_3_ (AR, Aladdin) and 0.46 g Co(NO_3_)_2_·6H_2_O (AR, Aladdin) were dissolved in a mixed solvent containing 140 mL deionized water and 20 mL ethylene glycol at 80 °C under vigorous stirring to form a homogeneous solution. Then, 2.8 g C_6_H_12_N_4_ (HMT) (AR, Aladdin) was added to the above solution under stirring for 4 h. With the assistance of HMT, the larger crystal nucleus continuously absorbs the mass produced by the dissolution of the smaller crystal nucleus and then grows, which is caused by the descend of interface energy of the total surface of the particle phase [25]. Next, the solution was filtrated and washed several times with deionized water and ethanol to collect a precipitate. The precipitate Cwas dried in an oven at 80 ℃ for 12 h to obtain the precursor. Finally, the Co_2_VO_4_ precursor was calcined in a tubular furnace at 350 ℃ for 4 h under Ar/H_2_ (90%/10%) atmosphere, which mainly removed water in the precursor to form the porous Co_2_VO_4_ nanodisk. The Schematic illustration of PCVO ND synthesis is shown in Fig. 1.

### Characterization

The crystalline characteristic of as-prepared samples was identified by X-ray diffractometer (XRD, Rigaku MiniFlexll) with Cu Kα radiation (λ = 0.15406 nm) at a scanning angle (2θ) range of 10° to 90°. X-ray photoelectron spectra (XPS, Thermo Scientific K-Alpha) measurements were performed using an Al Kα X-ray source. Nitrogen adsorption–desorption isotherms were conducted by a Quantachrome instruments Autosorb-IQ3 system. The morphology and surface details of PCVO ND were analyzed by scanning electron microscopy (SEM, JEOL JSM-7800F Field Emission) and transmission electron microscopy (TEM, JEOL JEM, 1011).

### Electrochemical Measurements

The electrodes were prepared by mixing 70 wt.% active material (PCVO ND or graphite or Li_4_Ti_5_O_12_ or Si), 20 wt.% acetylene black, and 10 wt.% polyvinylidene fluoride (PVDF) in N-methylpyrrolidinone (NMP) to form a slurry. The slurry was cast on copper foil and dried in a vacuum oven at 80 ℃ for 12 h. The active material mass loading on each electrode for graphite, Li_4_Ti_5_O_12_, Si, and PCVO ND is around 0.6 mg cm^−2^. The CR2025 coin-type half cells were assembled in an Ar-filled glovebox using the as-prepared electrode as the working electrode, lithium foil as the counter electrode, and a Cellgard 2400 microporous membrane as the separator. 1 M LiPF_6_ dissolved in ethylene carbonate (EC) and dimethyl carbonate (DEC) (1:1, in volume) with 5 wt.% fluoroethylene carbonate (FEC) was used as the electrolyte. The GITT test was carried out on a LAND battery-test instrument (CT2001A) to obtain the Li^+^ diffusion coefficient of PCVO ND. Cyclic voltammetry (CV) curves between 0 V and 3.0 V with a scan rate of 0.2 mV s^−1^ were tested by an electrochemical workstation (CHI-660D). Electrochemical impedance spectroscopy (EIS) curves were recorded by CHI-660D electrochemical workstation, and the frequency range is 100 kHz to 0.01 Hz. The fast-charging performance of PCVO ND was measured at 4 C and 10 C using a LAND- CT2001A system (1 C = 1000 mA g^−1^). For comparison, graphite, Li_4_Ti_5_O_12_, Si was also measured at 400 mA g^−1^ and 4000 mA g^−1^. Besides, the lithium-ion full cell was assembled using LiCoO_2_ and PCVO ND as cathode and anode, respectively. The cathode was fabricated by mixing 70 wt.% LiCoO_2_, 20 wt.% acetylene black, and 10 wt.% PVDF in NMP to construct slurry and then spread on Al foil. The PCVO ND was first pre-activated in a half cell to form a stable solid electrolyte interface (SEI) layer and then taken out as anodes for the LIB full cell. The electrolyte and separator in the full cell were identical to those in a half cell.

## Results and Discussion

### Theoretical Calculations

**Fig. 2:**
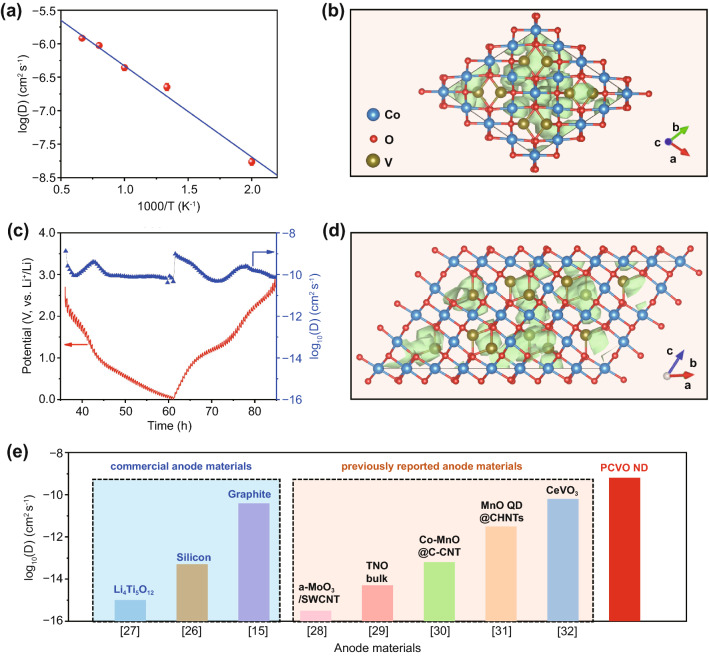
**a** Arrhenius plot of overall diffusion coefficient, where the error bar corresponds to statistical uncertainty in the fitting of the mean square displacement to time curve. **b** Top view and **d** side view of isosurfaces of lithium ion probability density during an AIMD simulation. **c** GITT curve and corresponding Li^+^ diffusion coefficient of PCVO ND. **e** The comparison of Li^+^ diffusion coefficient of PCVO ND with commercial anode materials and other previously reported anode materials

To design fast-charging high-energy–density LIBs, high capacity, fast lithium diffusion, suitable working potential, and high cycling stability are four essential parameters for electrode materials. Co_2_VO4 exhibits several advantages in high-energy–density LIBs, such as high capacity (~ 1000 mAh g-1), relatively low yet safe lithiation potential (~ 0.65 V vs. Li^+^/Li), and excellent electrochemical stability [16]. Thus, we hypothesized that Co_2_VO_4_ could be an attractive fast-charging anode material for LIBs. To prove it, we first calculated the Li^+^ diffusion coefficient (*D*_Li_) of Co_2_VO_4_ for all paths found by the AIMD simulations. The *D*_Li_ was calculated based on the averaged mean square displacement of Li ions over time. Convergence of *D*_Li_ was achieved by MD simulations of 50 ps approximately. The Arrhenius plot for the variety of *D*_Li_ at temperatures from 500 to 1500 K is shown in Fig. 2a. The extrapolated *D*_Li_ at 300 K was 3.15 × 10^–10^ cm^2^ s^−1^ which was 1 ~ 6 orders of magnitude higher than that of commercial graphite (4.0 × 10^–11^ cm^2^ s^−1^) [26], Li_4_Ti_5_O_12_ (1.0 × 10^–15^ cm^2^ s^−1^) [27], and Si (4.60 × 10^–14^ cm^2^ s^−1^) [15], theoretically proving Co_2_VO_4_ a promising anode material for fast-charging LIBs. The trajectory of Li^+^ diffusion was described by its time-averaged spatial occupancy probability in the crystal structure, of which top view and side view is displayed in Fig. 2b, d. The probability densities of Li ions were localized in the space between pairs of point-sharing CoO_6_ octahedra and VO_4_ tetrahedra. Migration could occur via the channels connecting octahedrally coordinated interstitial sites along subdiagonal of *a*, *b,* or *c* axes, where each of interstitial site is surrounded by 6 O ions.

Besides the inherent property of anode materials, material structure design is also important for achieving good fast-charging performance. The porous nanodisk structural design can reduce the electron and ion diffusion length at the electrode level, further accelerating the electrochemical reactions. Thus, we designed a hexagonal porous Co_2_VO_4_ nanodisk (PCVO ND) structure and then investigated *D*_Li_ of our PCVO ND experimentally by galvanostatic intermittent titration technique (GITT) with a pulse current density of 100 mA g^−1^ for 10 min between 20 min rest intervals (Fig. 2c). The *D*_Li_ can be calculated by the following equation [27]:

3 $$D = \frac{{4L^{2} }}{\pi \tau }\left( {\frac{{\Delta E_{S} }}{{\Delta E_{t} }}} \right)^{2}$$

where *L* is lithium-ion diffusion length, *t* is the duration of the current pulse, and *τ*, *ΔE*_*S*_, and *ΔE*_*t*_ are the galvanic titration, voltage change between steps, and voltage change during the pulse period, respectively. Based on GITT measurement and Eq. (3), the average *D*_Li_ of PCVO was 6.95 × 10^–10^ cm^2^ s^−1^, showing the same order of magnitude that AIMD simulations have achieved. The *D*_Li_ of our PCVO ND was higher than that of commercial anode materials (graphite, Li_4_Ti_5_O_12_, and Si) and higher than that of previously reported fast-charging anode materials [28–32] (Fig. 2e), experimentally confirming PCVO ND's feasibility in fast-charging high-energy–density LIBs. These detailed *D*_Li_ are summarized in Table S1.

### Structure and Morphology of PCVO ND

**Fig. 3:**
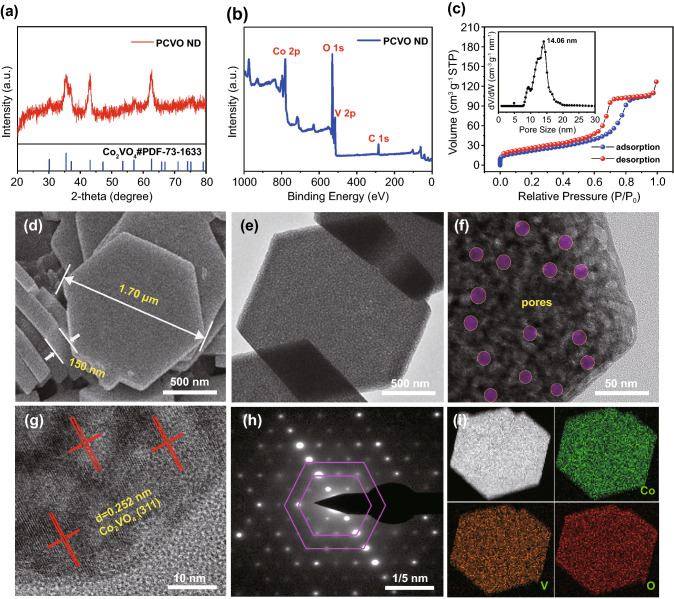
**a** XRD pattern of PCVO ND. **b** XPS full survey spectrum of PCVO ND. **c** Nitrogen adsorption and desorption isotherms of PCVO ND (Inset is the corresponding pore size distributions). **d** FESEM image of PCVO ND. **e** and **f** TEM images. **g** HRTEM image. **h** SAED pattern. **i** STEM and the corresponding elemental mapping images indicating the homogeneous distribution of all three elements of Co, V and O

Figure 3a shows the XRD pattern of PCVO ND. The diffraction peaks at 2θ of 30.1°, 35.5°, 37.1°, 43.1°, 47.2°, 53.5°, 57.0°, and 62.6° were indexed to the (220), (311), (222), (400), (331), (422), (511), and (440) diffraction planes of spinel Co_2_VO_4_ (JCPDS No.73–1633) [33], indicating PCVO ND has high crystallinity. PCVO ND's face-centered cubic spinel structure was displayed in Fig. S1a, composing of Fd-3 m space group and cubic cells. Both Co and V atoms occupy the tetrahedral and octahedral crystallographic sites in the crystal structure of Co_2_VO_4_. The two adjacent octahedral sites were formed as a chain by sharing their two oxygen edge-shared corners. The solitary octahedral sites in two different chains were connected to the two separate tetrahedral sites by cross-linking their individual oxygen corners to complete the entire cubic structure. The stable crystal structure was beneficial to improve cycling stability. To investigate PCVO ND's chemical information, XPS measurement was carried out and the result is shown in Fig. 3b. The Co, V, and O elements can be clearly indicated in the full survey spectrum of PCVO ND. Figure S1b shows the Co 2p spectra in which two intense peaks at 780.1 eV and 796.5 eV can be assigned to Co^2+^ [35]. Figure S1c displays the high-resolution spectra of V 2p in which two peaks at 516.4 eV and 523.7 eV originated from V 2p_3/2_ and V 2p_1/2_, respectively. The two peaks were ascribed to V^4+^ [36]. The O 1 s signal could be fitted into three oxygen components around 529.9, 530.8, and 531.85 eV (Fig. S1d), which were attributed to the metal–oxygen bonds (M–O), hydroxyl species of surface adsorbed water molecule (M-OH), and oxygen ions in low coordination (O_L_), respectively [35, 36].

The Brunner-Emmet-Teller (BET) and Barrett-Joyner-Halenda (BJH) methods were used to study the porous structure of the synthesized PCVO ND, and the results are shown in Fig. 3c. From Fig. 3c, the nitrogen adsorption–desorption isotherm of PCVO ND matched to a typical type IV curve [37], confirming PCVO ND mesoporous structure. And the H3 type hysteresis indicated PCVO ND disk-like structure. The BET specific surface area of PCVO ND was 74.57 m^2^ g^−1^, much higher than that of other cobalt vanadates (Table S2). The large specific surface area of PCVO ND is beneficial to contact between electrode and electrolyte and can provide more reactive sites, promoting the electrode reaction kinetics. And the BJH analysis revealed that PCVO ND presented a uniform and small pore size of 14.06 nm among our PCVO ND sample. The numerous mesopores in PCVO ND shorten the transmission distance of Li^+^, improving the kinetics of the electrode reaction. Moreover, the porous structure provides a certain space to alleviate the volume expansion problem of PCVO ND during the cycling, prolonging the electrode's cycle life.

The morphology, structure, and element distribution of PCVO ND were characterized by SEM and TEM. Figures 3d and S2a show the low-magnification SEM images of hexagonal Co_2_VO_4_ nanodisk with a width of ~ 0.15 μm and thickness of ~ 150 nm. The high magnification SEM image (Fig. S2b) revealed a rough surface of PCVO ND in which many pores exist, providing more reactive sites for active substances. Furthermore, the TEM images (Fig. 3e, f) confirmed the nanodisk morphology and mesoporous structure of PCVO ND. The purple round areas presented pores in PCVO ND (Fig. 3f) and the mesopore size was about 15 nm, in agreement with BET results. High-resolution TEM image of PCVO ND (Fig. 3g) revealed a regular atomic arrangement and lattice spacing of 0.252 nm, corresponding to the crystal planes of (311) of spinel Co_2_VO_4_ [33]. The selected area electron diffraction (SAED) (Fig. 3h) showed well-defined diffraction spots with hexagonal symmetry, indicating the polycrystalline nature of Co_2_VO_4_. Figure 3i showed a low-magnification high-angle annular dark-field (HAADF) STEM image of PCVO ND and corresponding element mapping. Energy-dispersive spectroscopy (EDS) mapping images revealed that PCVO ND was composed of Co, V, and O elements, which were homogeneously distributed in the nanodisk. According to the EDS pattern (Fig. S3), the mass fractions of Co, V, and O atoms were 49.72 wt.%, 21.93 wt.%, and 28.35 wt.%, respectively. By calculating based on relative atomic mass, PCVO ND exhibited the mole ratio of Co/V/O was 1.96: 1: 4.12, close to the stoichiometry of Co_2_VO_4_. These results demonstrated that the porous Co_2_VO_4_ nanodisk was successfully synthesized.

### Electrochemical Properties of PCVO ND

**Fig. 4:**
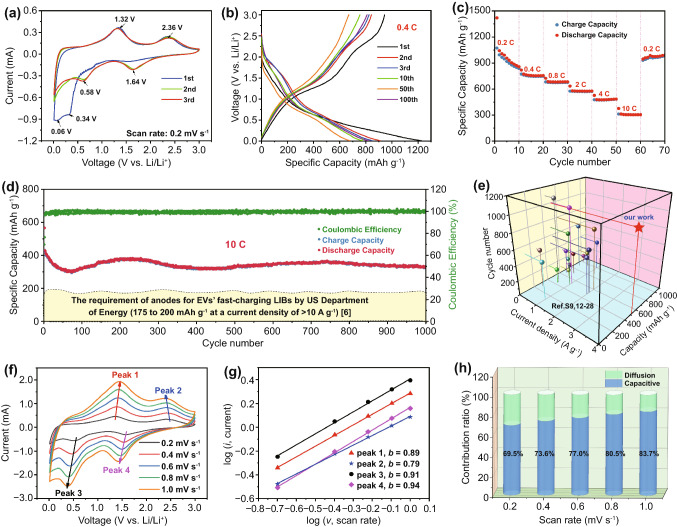
Electrochemical properties of PCVO ND. **a** CV curves. **b** Galvanostatic discharge and charge curves at 0.4 C. **c** Rate performance. **d** Cycling performance at 10 C. **e** A comparison of electrochemical properties. **f** CV curves at different scan rates. **g** Log(*i*) versus log(*v*) plots at specific peaks. **h** Contribution ratio of the capacitive and diffusion-controlled capacities at various scan rates (1 C=1000 mA g^−1^)

Cyclic voltammetry (CV) was measured to evaluate the lithium storage mechanism of PCVO ND. The initial three CV curves of PCVO ND measured at a scan rate of 0.2 mV s^−1^ between 0 and 3 V (vs. Li^+^/Li) are shown in Fig. 4a. It can be found that the cathodic behavior of the first cycle was different from that of subsequent cycles. The first cycle only showed two cathodic peaks around 0.06 and 0.34 V. The cathodic peak around 0.06 V corresponded to the formation of SEI film and the peak at 0.34 V was related to the transformation from Co_2_VO_4_ to CoO and Li_x_VO_2_, as shown in reaction Eq. (4) [17]. These peaks disappeared in the subsequent cycles because the formation of SEI had been complicated and the transformation in reaction Eq. (4) only occurred in the first cycle. In subsequent cycles, two new cathodic peaks at 1.64 and 0.58 V appeared, corresponding to the transformation from CoO to Co, Li_2_O and the intertcalation of Li^+^ into Li_x_VO_2_, as shown in reaction Eq. (5) [17]. In the delithiation process, PCVO ND showed two oxidation peaks at 1.32 and 2.36 V due to the oxidzation of Co and the extraction of Li^+^ from Li_x+y_VO_2_. The CV cures overlapped well, apart from the first cycle, manifesting the remarkable stability of PCVO ND.

4 $$Co_{2} VO_{4} + xLi^{ + } + xe^{ - } \to \, 2CoO \, + \, Li_{x} VO_{2}$$

5 $$CoO \, + \, Li_{x} VO_{2} + \, \left( {y + 2} \right) \, Li^{ + } + \left( {y + 2} \right) \, e^{ - } \leftrightarrow Co \, + \, Li_{2} O \, + Li_{x + y} VO_{2}$$

The PCVO ND was measured at 0.4 C within the voltage range of 0.01–3.0 V (vs. Li^+^/Li). Figure 4b displays PCVO ND's galvanostatic discharge–charge curves of the 1st, 2nd, 3rd, 10th, 50th, and 100th. Each discharge curve existed discharge platform in 0.65 V, consistent with CV results. The 2nd, 3rd, 10th, 50th, and 100th discharge and charge voltage curves almost overlap, revealing high reversibility. The discharge and charge capacity in the first cycle were 1227.0 and 944.1 mAh g^−1^, with an initial coulombic efficiency of 76.9%. The high discharge capacity in the first cycle is due to the formation of the SEI film. Figure S4 shows the cycling performance of PCVO ND at 0.4 C. PCVO ND displayed a high initial reversible capacity of 911.0 mAh g^−1^. The capacity showed a slight decrease before the 50th cycle and then increased slightly in the subsequent cycles. The capacity was recovered to 817.9 mAh g^−1^ after 100 cycles, corresponding to a high capacity retention of 89.8%, suggesting high cycling stability. Similar results have also been reported for the electrochemical performance of transition metal oxides in the literature [38, 39], which may be due to the continuous activation and improvement of Li^+^ ion accessibility with cycling. The exceptional electrochemical performance of PCVO ND electrode at 0.4 C was verified by electrochemical impedance spectroscopy (EIS) test. Figure S5a, b showed Nyquist plots and equivalent circuit model of PCVO ND electrode before cycling and after 1, 10, 50, and 100 cycles. R_1_ and R_2_ represent internal resistance, including electrolyte resistance, the internal resistance of active material, and contact resistance between the current collector and active material, while R_3_ represents the charge transfer resistance. The internal resistance of the PCVO ND electrode before cycling is much larger than that for the following cycles, mainly due to the much-enhanced ion and electron transfer in the electrode. Subsequently, there was little change in the impedance of the PCVO ND electrode, indicating low transfer resistance and good stability of PCVO ND.

Above good electrochemical performances (suitable lithiation potential, high capacity, high cycling stability) along with high Li^+^ diffusion coefficient from both theoretical and experimental results in Fig. 2a, c indicated that PCVO ND has excellent potential in fast-charging LIBs. Thus PCVO ND was tested at high rates. Increasing the rate from 0.2, 0.4, 0.8, 2, 4 to 10 C (Fig. 4c), PCVO ND anode delivered average specific discharge capacities of 857.7, 755.5, 684.6, 575.9, 485.3, and 305.2 mAh g^−1^, respectively. Meanwhile, returning the rate from 10 to 0.2 C, PCVO ND anode's specific discharge capacity recovered to 1021.3 mAh g^−1^, exhibiting good rate performance and excellent reversibility. The high fast-charging capability of anodes after long-term cycles is of practical importance for applications in EVs. Thus PCVO ND was further tested at 10 C for 1000 cycles (Fig. 4d). Notably, the capacity decreased during the first 70 cycles and gradually increased in the subsequent cycles. This phenomenon was observed in many transition oxides. The rapid capacity decrease is resulted from the conversion reactions, generated volume expansion and formation of SEI layer [16, 25, 34, 42]. The subsequent capacity increase might be attributed to the increased crystallinity of active materials and gradual activation progress during the cycle [17, 36, 38]. Figure 4d showed that PCVO ND exhibited excellent fast-charging capacity (a high average capacity of 344.3 mAh g^−1^ at 10 C for 1000 cycles) and outstanding long-term cycling stability (only 0.024% capacity loss per cycle at 10 C for 1000 cycles). A LIB with such PCVO ND anode can be fully charged within 5 min. Thus our PCVO ND met the requirement of anodes for fast-charging LIBs in EVs by the US Department of Energy and showed better fast-charging performance than previously reported fast-charging anodes, summarized in Fig. 4e and Table S3. Such excellent fast-charging performance could be attributed to the following reasons: (i) The various valence states of V and Co element and the combined intercalation and conversion reaction mechanism lead to a high theoretical capacity; (ii) The high Li^+^ diffusion coefficient of Co_2_VO_4_ can facilitate fast electrochemical reactions, and the porous nanodisk structural design can reduce the electron and ion diffusion length, further enhancing the electrochemical kinetics; (iii) Numerous internal pores of PCVO ND and uniform nanoscale pore size buffer the volume change, and the close contact between electrode and electrolyte by PCVO ND' large specific surface area stabilize the SEI film, leading to superior long-term cycling stability of PCVO ND at a high rate.

To further investigate PCVO ND's stability, the morphologies of PCVO ND before and after 1000 cycles were examined. Figure S6a–d showed the SEM images of the PCVO ND electrode before and after 1000 cycles at 10 C. The PCVO ND exhibits a high structural stability and still maintains a hexagonal structure after 1000 cycles (Fig. S6c, d), confirming the excellent long-term cycle stability of PCVO ND. The structure of the PCVO ND after 1000 cycles was also investigated to conform the stability of PCVO ND. The XRD pattern of PCVO ND after 1000 cycles at 10 C is shown in Fig. S6e. Compared to the pristine PCVO ND, the peaks of Co_2_VO_4_ were absent in the electrode after 1000 cycles. Meanwhile, two new peaks at 44.3° and 65.7° corresponded to the (015) and (110) diffraction planes of LiVO_2_, respectively [17]. The HRTEM image of PCVO ND after 1000 cycles at 10 C (Fig. S6f) confirmed the exist of LiVO_2_ (lattice spacing of 0.201 nm). And the lattice spacing of 0.151 nm corresponded to the (220) of CoO [17]. The XRD and HRTEM results demonstrated that PCVO ND still followed the reaction mechanism: CoO + Li_*x*_VO_2_ + (y + 2) Li^+^  + (y + 2) e^−^ ⟷ Co + Li_2_O + Li_x+y_VO_2_ after 1000 cycles, further confirming the stability of PCVO ND cycled at a high rate.

To get deep insight into the excellent lithium storage kinetics of PCVO ND, CV measurements were carried at different scan rates ranging from 0.2 to 1.0 mV s^−1^ (Fig. 4f). The shapes of the characteristic peaks were similar with increasing scan rates. The kinetics of charge storage mechanism could be analyzed by the relationship between current (*i*) and scan rate (*v*) which can be described by the following equation:

6 $$i = av^{b}$$

In this equation, a and b are adjustable parameters. The value of b ranges from 0.5 to 1.0 (0.5 for a diffusion-controlled charge storage mechanism and 1.0 for a capacitive charge storage mechanism [40]. The values of b can be calculated by the fitting the log (*i*) versus log (*v*). As shown in Fig. 4g, the values of b were calculated to be 0.89, 0.79, 0.91, and 0.94 for peaks 1, 2, 3, and 4, respectively. The values of b were in the range of 0.8–1.0, suggesting a high pseudocapacitive contribution of lithium storage in PCVO ND electrode. This was further confirmed by quantifying the capacitive (k_1_*v*) and diffusion-controlled storage contribution (k_2_*v*^1/2^) using the following formula:

7 $$i = k_{1} v + k_{2} v^{1/2}$$

8 $$i/v^{1/2} = k_{1} v^{1/2} + k_{2}$$

As shown in Fig. S7, a 77.0% contribution was obtained from capacitive charge storage when scan rate was 0.6 mV s^−1^. A summary of these contributions by both components was plotted at different scan rate (Fig. 4h). The capacitive contribution increased from 69.5% to 83.7% with scan rate increasing. PCVO ND involves a pseudocapacitive charge storage mechanism concluded from above analysis. The high pseudocapacitive storage contribution might originate from porous structure of electrode, which contributed to increase surface area and enhance charge transfer kinetics.

### Comparison with Commercial Anode Materials

**Fig. 5:**
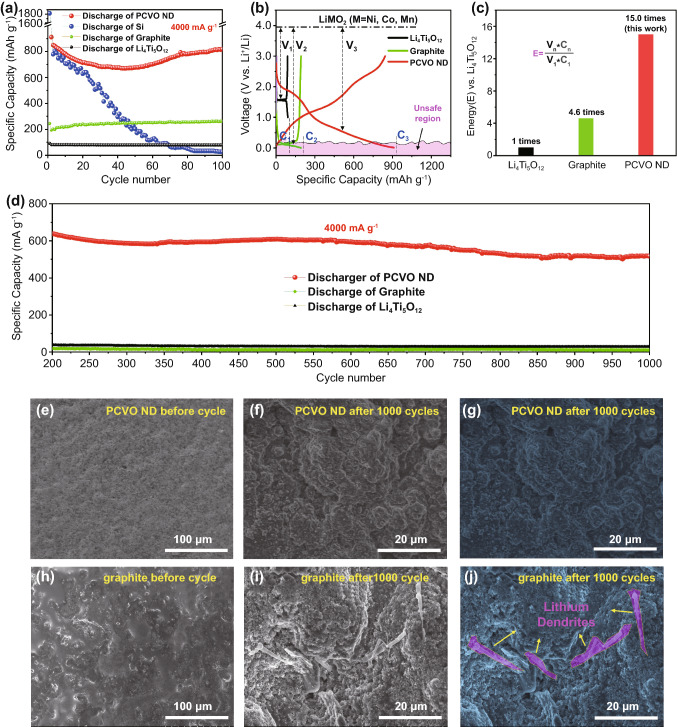
**a** The cycling performance of PCVO ND and commercial graphite, Si and Li_4_Ti_5_O_12_ anode materials at 400 mA g^−1^. **b** The potential versus capacity plots of PCVO ND, commercial graphite, and Li_4_Ti_5_O_12_ at 400 mA g^−1^. **c** The energy density comparison of PCVO ND, commercial graphite, and Li_4_Ti_5_O_12_. **d** The long-term cycling performance of PCVO ND, Li_4_Ti_5_O_12_, and graphite at 4000 mA g^−1^ from the 200th to 1000th cycle. **e** SEM image of PCVO ND electrode before cycling. **f ** and **g ** SEM images of PCVO ND electrode after 1000 cycles at 4000 mA g^−1^. **h** SEM image of graphite electrode before cycling. **i** and **j** SEM images of graphite electrode after 1000 cycles at 4000 mA g^−1^

In order to evaluate the practical application, the electrochemical performances of PCVO ND were compared with that of anodes in commercial LIBs. Figure 5a shows the cycling performance of PCVO ND, graphite, Si, and Li_4_Ti_5_O_12_ at 400 mA g^−1^. It can be seen that the initial capacities of PCVO ND, graphite and Li_4_Ti_5_O_12_ were 1226.9, 245.5, and 91.9 mAh g^−1^ while they were 817.9, 262.7, and 79.4 mAh g^−1^ after 100 cycles. Their cycling performances were excellent, except Si showed a fast capacity decay (1819.7 to 27.2 mAh g^−1^). Thus, Si anode was not further studied in this paper. Figure 5b shows the potential versus capacity plots of PCVO ND, graphite and Li_4_Ti_5_O_12_ at 400 mA g^−1^. Li_4_Ti_5_O_12_ displayed the lowest discharge capacity of 164.1 mAh g^−1^ and intercalated Li^+^ at the highest potential (~ 1.55 V vs. Li^+^/Li). Such low capacity, along with high operation voltage, sacrificed the cell voltage and cell energy seriously. In contrast, PCVO ND showed the highest discharge capacity of 910.9 mAh g^−1^. And the insertion of Li^+^ into PCVO ND occurred at a lower potential (~ 0.65 V vs. Li^+^/Li). The energy density formula in Fig. 5c is defined as:

9 $$E = \frac{Cn*Vn}{{C1*{\text{V1}}}}$$

where the *E* is the energy density relative to the LTO anode, *C*_*n*_ is the specific capacity of different anodes, *V*_*n*_ is the voltage difference between discharge platform and commercial 4 V cathode materials (*n* = 1, 2, 3, 1 means to LTO, 2 means to graphite, 3 means to CVO) [41].

The estimated energy density of PCVO ND (regarding both potential and capacity) was 15 orders of magnitude higher than that of Li_4_Ti_5_O_12_ if the two are coupled with a typical 4 V cathode (Fig. 5c). Thus, Li_4_Ti_5_O_12_ is not suitable in high-energy–density fast-charging LIBs.

For graphite, it intercalated Li^+^ at the lowest voltage (~ 0.1 V vs. Li^+^/Li) close to that of the Li-plating, which resulted in a safety risk due to high surface Li-plating (Li dendrite, a potential cause of short circuits). In order to compare the PCVO ND's safety with graphite at high rates, the PCVO ND and graphite anodes were charged and discharged at 4000 mA g^−1^ for 1000 cycles. Both PCVO ND and graphite anodes showed high cycling stability (Figs. 5d and S8). Nevertheless, PCVO ND anode displayed a high average capacity of 579.2 mAh g^−1^, while graphite anode showed an average capacity of only 12.1 mAh g^−1^ (Fig. 5d). SEM images of PCVO ND and graphite anodes before and after 1000 cycles are shown in Fig. 5e-j. There were no visible Li dendrites in PCVO ND electrode after 1000 cycles (Fig. 5f, g). Li dendrites (with a wire-like shape) were clearly found in graphite electrodes (Fig. 5i, j). Besides, the EIS of PCVO ND electrode cycled at 4000 mA g^−1^ from before cycling to after 1000 cycles was also tested. The results are shown in Fig. S9. The impedance had minor differences between the 100th and 1000th cycles, confirming that the Li dendrites have not been formed after 1000 cycles. These results confirmed the commercial feasibility of PCVO ND in safe, fast-charging, high-energy–density LIBs.

### Full Cell Performance of PCVO ND

**Fig. 6:**
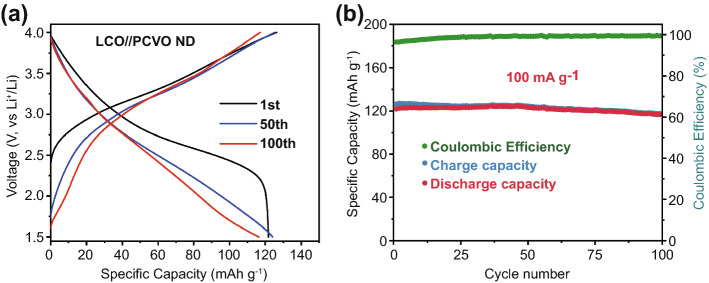
**a** Galvanostatic charge/discharge curves for the 1st, 50th, and 100th cycles at a current density of 100 mA g^−1^ over the potential range of 1.5–4.0 V. **b** Cycling performance of the LiCoO_2_// PCVO ND full cell at a current density of 100 mA g^−1^

According to the outstanding performance of the Li//PCVO ND half cells, we investigated the full cell performance using PCVO ND as anode and commercial LiCoO_2_ materials as the cathode. According to the principle of capacity balance, the mass ratio of PCVO ND to LiCoO_2_ was controlled to be about 1: 6. The half-cell battery with the active material was pre-activated to form a stable SEI layer and then taken out to fabricate the full cell. Figure 6a presents the full cell voltage profiles after the 1st, 50th, and 100th cycles at the voltage window of 1.5–4.0 V at 100 mA g^−1^. The initial charge and discharge capacities are 126.4 and 121.8 mAh g^−1^, respectively, exhibiting a high coulombic efficiency of 96.3%. Besides, the cycling performance of lithium-ion full cell is shown in Fig. 6b. The full cell exhibits a good discharge capacity of 116.4 mAh g^−1^ after 100 cycles with a capacity retention of 95.5%. From these results, it is evident that the LiCoO_2_//PCVO ND full cell exhibits outstanding performance in terms of reversible capacity, stable cycling behavior and high coulombic efficiency, confirming the potential of the PCVO ND as an anode material for LIBs.

## Conclusions

Our studies showed that Co_2_VO_4_ was a promising anode material for high-energy–density fast-charging LIBs due to its high capacity (~ 1000 mAh g^−1^), safe lithiation potential (~ 0.65 V vs. Li^+^/Li), high Li^+^ diffusion coefficient (6.95 × 10^–10^ cm^2^ s^−1^), and high cycling stability. In order to further enhance Li^+^ and electrons transfer, we designed a hexagonal porous Co_2_VO_4_ nanodisk (PCVO ND) structure, which features a high specific surface area of 74.57 m^2^ g^−1^ and numerous pores with a uniform pore size of 14 nm in PCVO ND. The porous nanodisk structural design can reduce the electron and ion diffusion length at the electrode level, further enhancing the electrochemical kinetics. Moreover, the close contact between electrode and electrolyte by the large specific surface area of PCVO ND stabilizes the SEI film, leading to superior fast-charging performance of PCVO ND. As a result, the PCVO ND showed a high initial reversible capacity of 911.0 mAh g^−1^ at 0.4 C, excellent fast-charging capacity (average capacity of 579.2 mAh g^−1^ at 4 C and 344.3 mAh g^−1^ at 10 C for 1000 cycles), outstanding long-term cycling stability (only 0.024% capacity loss per cycle at 10 C for 1000 cycles). Due to its superior fast-charging performance and simple preparation, the PCVO ND has high commercial feasibility in high-energy–density fast-charging LIBs.

## Supplementary Information

Below is the link to the electronic supplementary material.

Supplementary file1 (PDF 694 KB)
