# Supplementary material for: Porous Co2VO4 Nanodisk as a High-Energy and Fast-Charging Anode for Lithium-Ion Batteries
Source: Nanomicro Lett. 2021 Dec 2;14:5. doi: 10.1007/s40820-021-00758-5 (PMC8639887; doi:10.1007/s40820-021-00758-5)
Supplement: Supplementary file 1 — Supplementary file1 (PDF 694 KB) [file 40820_2021_758_MOESM1_ESM.pdf]

Supporting Information for

Porous Co_2VO_4 Nanodisk as a High-Energy and Fast-Charging Anode for Lithium-Ion Batteries

Jinghui Ren[1, #], Zhengyu Wang[2, 3, #], Peng Xu[1], Cong Wang[1], Fei Gao[1], Decheng Zhao[1], Shupei Liu[1], Han Yang[1], Di Wang[1], Chunming Niu[2], Yusong Zhu[1], Yutong Wu[1], Xiang Liu[1], Zhoulu Wang[1], Yi Zhang[1, *]

[1]School of Energy Sciences and Engineering, Nanjing Tech University, Nanjing 211816, P. R. China

[2]Center of Nanomaterials for Renewable Energy, State Key Laboratory of Electrical Insulation and Power Equipment, School of Electrical Engineering, Xi'an Jiaotong University, Xi'an 710054, P. R. China

[3]Department of Computational Materials Design, Max-Planck-Insitut für Eisenforschung GmbH, Max-Planck-Straße 1, 40237 Düsseldorf, Germany

#Jinghui Ren and Zhengyu Wang author have contributed equally to this work.

*Corresponding author. E-mail: zhangy@njtech.edu.cn (Yi Zhang)

Supplementary Figures

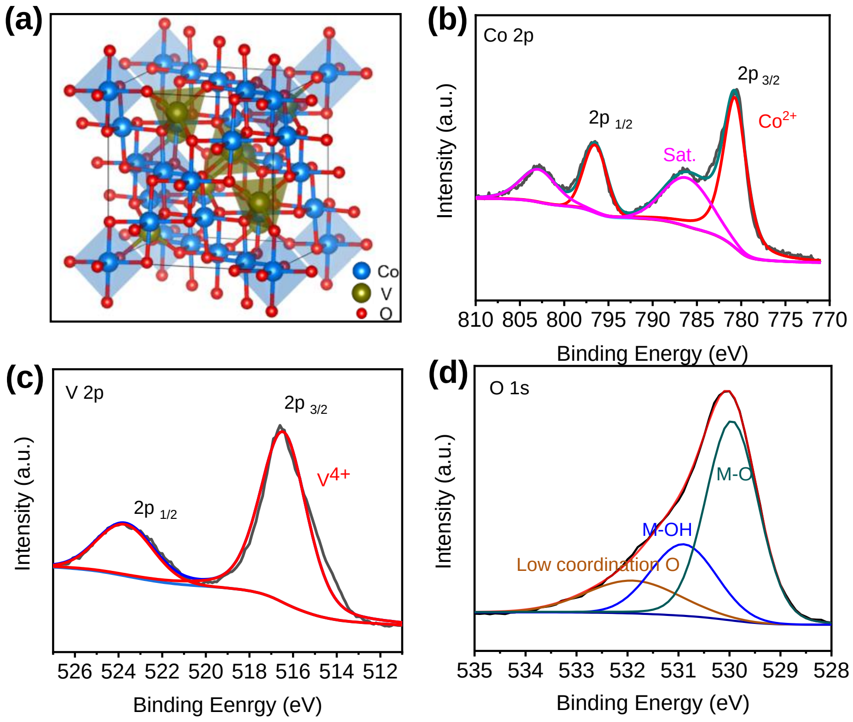

Fig. S1 a Crystal structure of Co_2VO_4. Core-level XPS spectra of **b** Co, **c** V, and **d** O elements in the hybrid material

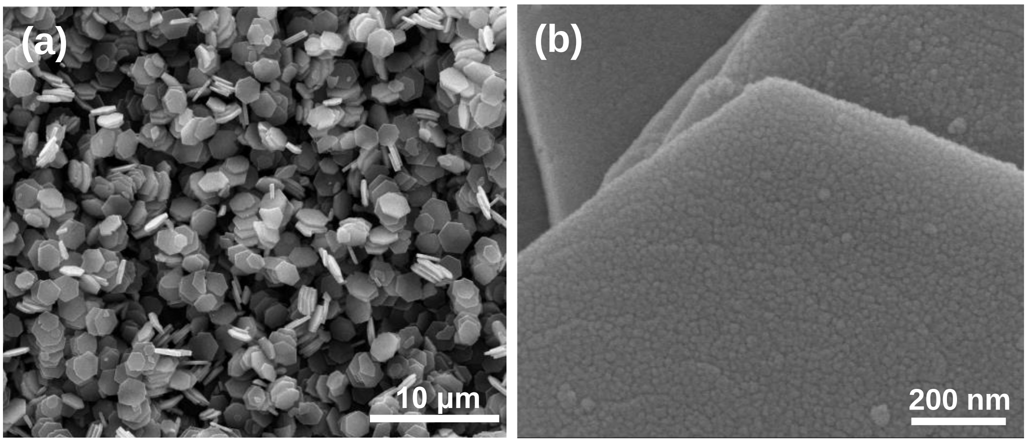

Fig. S2 SEM images of PCVO ND with different magnifications

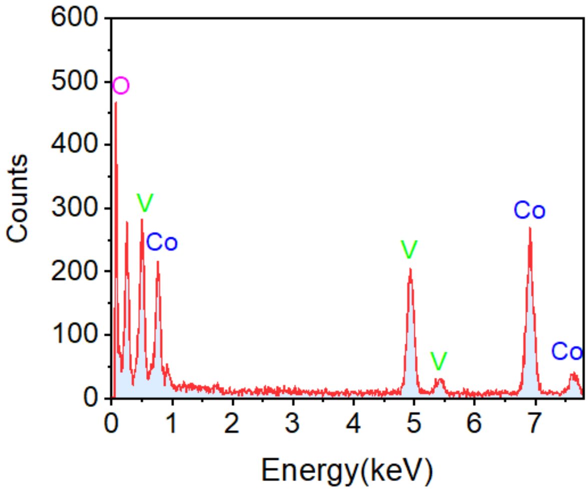

Fig. S3 EDS spectrum of PCVO ND

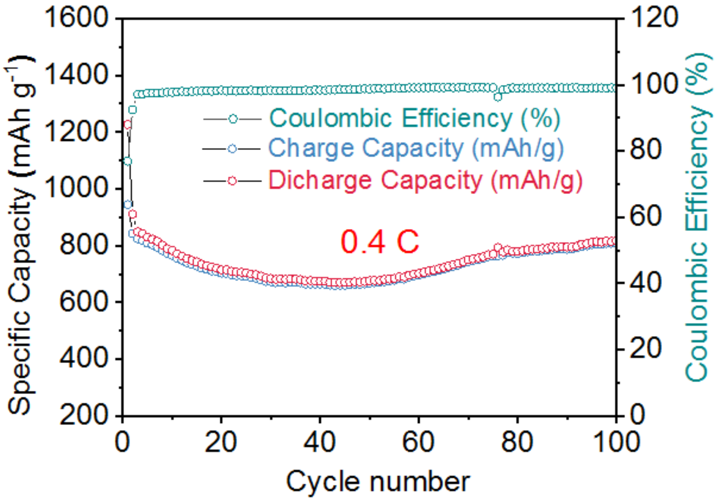

Fig. S4 Cycling performance of PCVO ND at 0.4 C

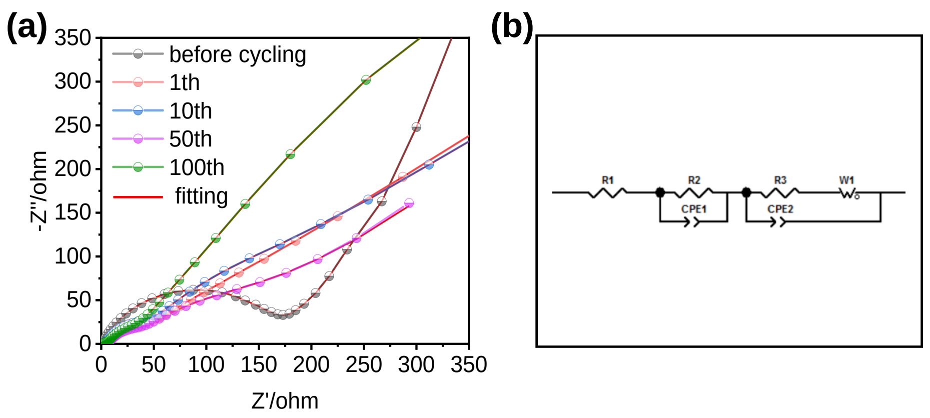

Fig. S5 a The Nyquist plots of the PCVO ND electrode at 0.4 C before cycling and after different cycles. **b** Selected equivalent circuit model

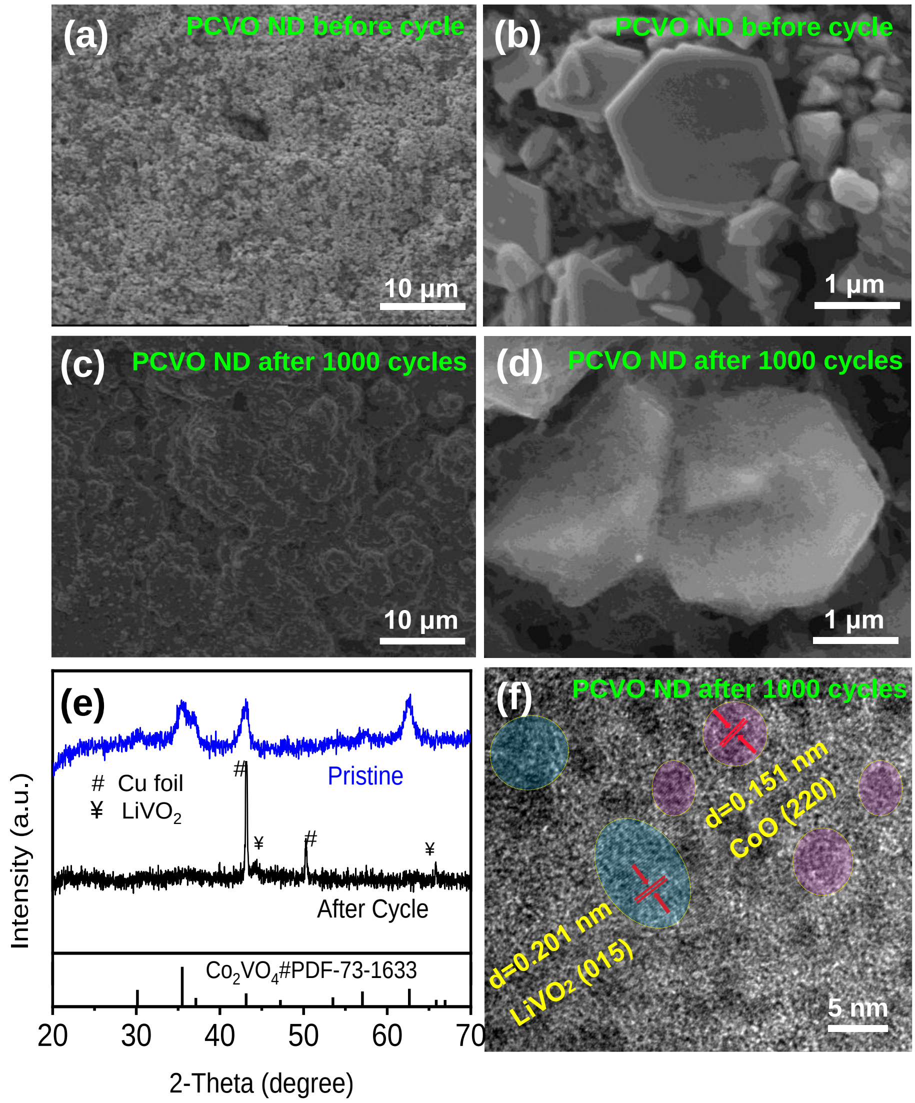

Fig. S6 SEM images of PCVO ND electrode: **a** and **b** Before cycle. **c** and **d** After 1000 cycles at 10 C. **e** The XRD patterns of PCVO ND electrodes before cycle and after 1000 cycles at 10 C. **f** HRTEM image of PCVO ND after 1000 cycles at 10 C

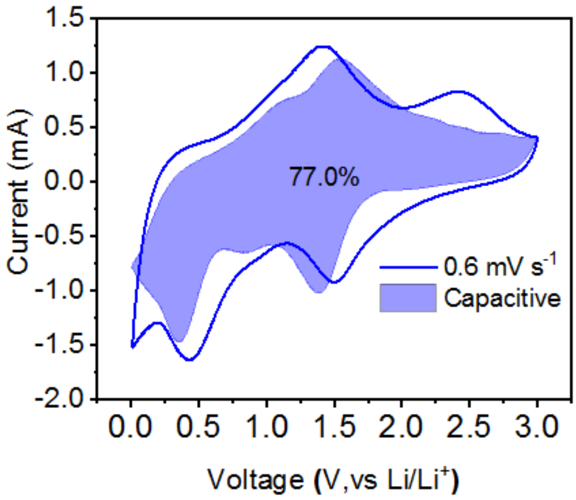

Fig. S7 Capacitive (blue) contribution of PCVO ND electrode at the scan rate of 0.6 mV s^{-1}

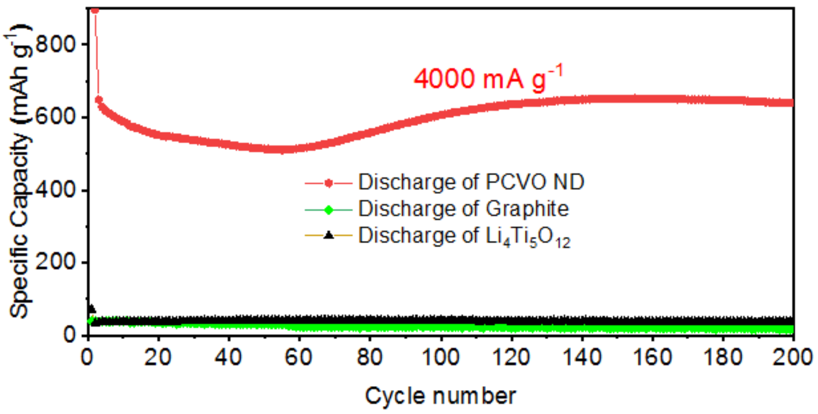

Fig. S8 The long-term cycling performance of PCVO ND, $Li_4Ti_5O_{12}$, and graphite at 4000 mA g^{-1} from the 1st to 200th cycle

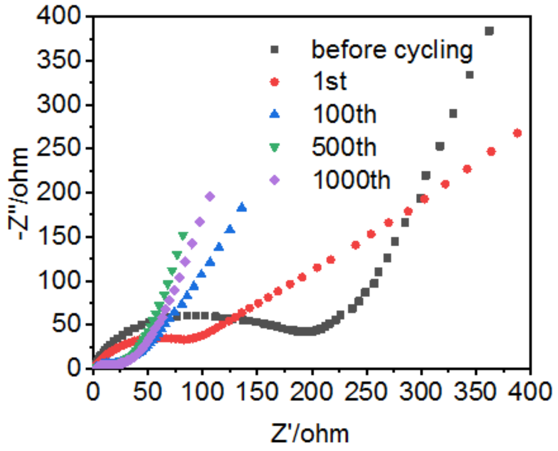

Fig. S9 The Nyquist plots of the PCVO ND electrode before cycling and after the 1st, 100th, 500th, 1000th cycle at 4000 mA g^{-1}

Table S1 Comparison of the Li^+ diffusion coefficient of our PCVO ND with that of the previously reported LIB anodes

Anode materials	Average D_{Li^+} (cm^2 S^{-1})	Refs.
Commercial graphite	4.0×10^{-11}	[S1]
Commercial $Li_4Ti_5O_{12}$	1.0×10^{-15}	[S2]
Commercial silicon	4.60×10^{-14}	[S3]
α-MoO_3/SWCNH	3.40×10^{-16}	[S4]
TNO bulk	4.70×10^{-15}	[S5]
Co-MnO@C-CNTs	6.93×10^{-14}	[S6]
MnOQD@CHNTs	2.98×10^{-12}	[S7]
$CeVO_3$	6.55×10^{-11}	[S8]
**PCVO ND**	**6.95×10^{-10}**	**Our work**

Table S2 Comparison of the surface area of PCVO ND with that of the previously reported other cobalt vanadates

Structure	Surface area (m^2 g^{-1})	Refs.
$Co_3V_2O_8$ Sponge Network	8.0	[S9]
$Co_3V_2O_8$ Multilayered Nanosheets	39.4	[S10]
Cobalt-vanadium bimetal-based nanoplates	29.2	[S11]
$Co_2V_2O_7$ hexagonal microplates	11.2	[S12]
**PCVO ND**	**74.57**	**Our work**

Table S3 Comparison of the electrochemical properties of our PCVO ND with that of the previously reported LIB anodes

Structure	Cycling stability	Refs.
$Co_3V_2O_8$ Sponge Network	501 mAh g^{-1} after 700 cycles at 1 A g^{-1}	[S9]
$Co_2V_2O_7$ hexagonal microplatelets	520 mAh g^{-1} after 580 cycles at 2 A g^{-1}	[S12]
Co_2VO_4@NC	993 mAh g^{-1} after 400 cycles at 1 A g^{-1}	[S13]
rGO@CoV PNSs	531.8 mAh g^{-1} after 1000 cycles at 1 A g^{-1}	[S14]
solid $Co_3V_2O_8$ micro-pencils	670 mAh g^{-1} after 330 cycles at 0.2 A g^{-1}	[S15]

CoV_2O_4	727.5 mAh g^{-1} after 100 cycles at 0.2 A g^{-1}	[S16]
$Co_3V_2O_8$ Hexagonal Pyramid	712 mAh g^{-1} after 300 cycles at 0.5 A g^{-1}	[S17]
hierarchical $Co_3V_2O_8$ microspheres	967.4 mAh g^{-1} after 200 cycles at 0.5 A g^{-1}	[S18]
rGO@$Co_3V_2O_8$ NP	899 mAh g^{-1} after 600 cycles at 0.2 A g^{-1}	[S19]
Macroporous CoV_2O_6 Nanosheet	702 mAh g^{-1} after 200 cycles at 0.2 A g^{-1}	[S20]
Fe_3O_4@C	601 mAh g^{-1} after 800 cycles at 2 A g^{-1}	[S21]
V_2O_3/porous N-doped carbon nanosheet	436 mAh g^{-1} after 200 cycles at 0.5 A g^{-1}	[S22]
N-doped carbon@Co_3O_4 nanoparticles	1017 mAh g^{-1} after 100cycles at 0.1 A g^{-1}	[S23]
CFMS	123 mAh g^{-1} after 500 cycles at 1 A g^{-1}	[S24]
Fe_2VO_4-PMP	483 mAh g^{-1} after 500 cycles at 2 A g^{-1}	[S25]
Carbon-coated MoO_3 nanofiber	623 mAh g^{-1} after 100 cycles at 0.5 A g^{-1}	[S26]
F doped Li_3VO_4	450 mAh g^{-1} after 1100 cycles at 0.5 A g^{-1}	[S27]
3D graphite nanoballs	282 mAh g^{-1} after 500 cycles at 0. 2 A g^{-1}	[S28]
**PCVO ND**	**519.4 mAh g^{-1} after 1000 cycles at 4 A g^{-1}**<br>**344.3 mAh g^{-1} after 1000 cycles at 10 A g^{-1}**	**Our work**

Supplementary References

[S1] W. Cai, C. Yan, Y.-X. Yao, L. Xu, R. Xu et al., Rapid lithium diffusion in order@disorder pathways for fast-charging graphite anodes. Small Struct. **1**, 2000010 (2020). https://doi.org/10.1002/sstr.202000010

[S2] C. Lin, B. Ding, Y. Xin, F. Cheng, M.O. Lai et al., Advanced electrochemical performance of $Li_4Ti_5O_{12}$-based materials for lithium-ion battery: synergistic effect of doping and compositing. J. Power Sources **248**, 1034-1041 (2014). https: //doi.org/10.1016/j.jpowsour.2013.09.120

[S3] Z. Xiao, C. Lei, C. Yu, X. Chen, Z. Zhu et al., Si@Si_3N_4@C composite with egg-like structure as high-performance anode material for lithium ion batteries. Energy Storage Mater. **24**, 565-573 (2020). https://doi.org/10.1016/j.ensm.2019.06.031

[S4] S.R. Sahu, V.R. Rikka, P. Haridoss, A. Chatterjee, R. Gopalan et al., A novel α-MoO_3/single-walled carbon nanohorns composite as high-performance anode

material for fast-charging lithium-ion battery. Adv. Energy Mater. **10**, 2001627 (2020). https://doi.org/10.1002/aenm.202001627

[S5] R. Tao, G. Yang, E.C. Self, J. Liang, J.R. Dunlap et al., Ionic liquid-directed nanoporous $TiNb_2O_7$ anodes with superior performance for fast-rechargeable lithium-ion batteries. Small **16**, e2001884 (2020). https://doi.org/10.1002/smll.202001884

[S6] Q. Sun, Z. Cao, J. Zhang, H. Cheng, J. Zhang et al., Metal catalyst to construct carbon nanotubes networks on metal oxide microparticles towards designing high-performance electrode for high-voltage lithium-ion batteries. Adv. Funct. Mater. **31**, 2009122 (2021). https://doi.org/10.1002/adfm.202009122

[S7] H. Li, L. Jiang, Q. Feng, Z. Huang, H. Zhou et al., Ultra-fast transfer and high storage of Li^+/Na^+ in MnO quantum dots@carbon hetero-nanotubes: appropriate quantum dots to improve the rate. Energy Storage Mater. **17**, 157-166 (2019). https://doi.org/10.1016/j.ensm.2018.07.021

[S8] S. Chen, H. Duan, L. Zhao, Y. Zhao, A. Gupta et al., Electrochemical performance and Li^+ insertion/extraction mechanism of carbon-coated cerium metavanadate as a novel anode for lithium-ion batteries. J. Power Sources **413**, 250-258 (2019). https://doi.org/10.1016/j.jpowsour.2018.12.053

[S9] V. Soundharrajan, B. Sambandam, J. Song, S. Kim, J. Jo, S. Kim et al., $Co_3V_2O_8$ sponge network morphology derived from metal-organic framework as an excellent lithium storage anode material. ACS Appl. Mater. Interfaces **8**, 8546-8553 (2016). https://doi.org/10.1021/acsami.6b01047

[S10] G. Yang, H. Cui, G. Yang, C. Wang, Self-assembly of $Co_3V_2O_8$ multilayered nanosheets controllable synthesis, excellent Li-storage properties, and investigation of electrochemical mechanism. ACS Nano **8**, 4474-4487 (2014). https://doi.org/10.1021/nn406449u

[S11] Y. Xiao, C. Tian, M. Tian, A. Wu, H. Yan et al., Cobalt-vanadium bimetal-based nanoplates for efficient overall water splitting. Sci. China Mater. **61**, 80-90 (2017). https://doi.org/10.1007/s40843-017-9113-1

[S12] F. Wu, C. Yu, W. Liu, T. Wang, J. Feng et al., Large-scale synthesis of $Co_2V_2O_7$ hexagonal microplatelets under ambient conditions for highly reversible lithium storage. J. Mater. Chem. A **3**, 16728-16736 (2015). https://doi.org/10.1039 /C5TA03106K

[S13] J. Liu, P. Zhang, D. Yu, K. Li, J. Wu et al., Hierarchical Co_2VO_4 yolk-shell microspheres confined by N-doped carbon layer as anode for high-rate lithium-ion batteries. J. Electroanal. Chem. **882**, 115027 (2021). https://doi.org/10.1016 /j.jelechem.2021.115027

[S14] S. Chandra Sekhar, G. Nagaraju, D. Narsimulu, B. Ramulu, S.K. Hussain et al., Graphene matrix sheathed metal vanadate porous nanospheres for

enhanced longevity and high-rate energy storage devices. ACS Appl. Mater. Interfaces **12**, 27074-27086 (2020). https://doi.org/10.1021/acsami.0c04170

[S15] J. Yang, M.Q. Wu, F. Gong, T.T. Feng et al., Facile and controllable synthesis of solid $Co_3V_2O_8$ micro-pencils as a highly efficient anode for Li-ion batteries. RSC Adv. **7**, 24418-24424 (2017). https://doi.org/10.1039/C7RA03118A

[S16] J.S. Lu, I.V.B. Maggay, W.R. Liu, CoV_2O_4: a novel anode material for lithium-ion batteries with excellent electrochemical performance. Chem. Commun. **54**, 3094-3097 (2018). https://doi.org/10.1039/C7CC09762J

[S17] Q. Zhang, J. Pei, G. Chen, C. Bie, D. Chen et al., $Co_3V_2O_8$ hexagonal pyramid with tunable inner structure as high performance anode materials for lithium ion battery. Electrochim. Acta **238**, 227-236 (2017). https://doi.org/10.1016/j.electacta.2017.04.013

[S18] H. Chai, Y.C. Wang, Y.C. Fang, Y. Lv, H. Dong et al., Low-cost synthesis of hierarchical $Co_3V_2O_8$ microspheres as high-performance anode materials for lithium-ion batteries. Chem. Eng. J. **326**, 587-593 (2017). https://doi.org /10.1016/j.cej.2017.05.162

[S19] G. Gao, S. Lu, B. Dong, Y. Xiang, K. Xi et al., Mesoporous $Co_3V_2O_8$ nanoparticles grown on reduced graphene oxide as a high-rate and long-life anode material for lithium-ion batteries. J. Mater. Chem. A **4**, 6264-6270 (2016). https://doi.org /10.1039/C5TA10719A

[S20] L. Zhang, K. Zhao, Y. Luo, Y. Dong, W. Xu et al., Acetylene black induced heterogeneous growth of macroporous CoV_2O_6 nanosheet for high-rate pseudocapacitive lithium-ion battery anode. ACS Appl. Mater. Interfaces **8**, 7139-7146 (2016). https://doi.org/10.1021/acsami.6b00596

[S21] Y. Liu, Y. Dai, X. Jiang, X. Li, Z. Yan et al., Fe_3O_4 quantum dots embedded in porous carbon microspheres for long-life lithium-ion batteries. Mater. Today Energy **12**, 269-276 (2019). https://doi.org/10.1016/j.mtener.2019.01.012

[S22] D. Zhang, G. Li, B. Li, J. Fan, X. Liu et al., A facile strategy to fabricate V_2O_3/Porous N-doped carbon nanosheet framework as high-performance anode for lithium-ion batteries. J. Alloys Compd. **789**, 288-294 (2019). https://doi.org/10.1016/j.jallcom.2019.02.251

[S23] F. Zheng, L. Wei, Synthesis of ultrafine Co_3O_4 nanoparticles encapsulated in nitrogen-doped porous carbon matrix as anodes for stable and long-life lithium ion battery. J. Alloys Compd. **790**, 955-962 (2019). https://doi.org/10.1016/j.jallcom.2019.03.212

[S24] Y. Feng, S. Chen, J. Wang, B. Lu, Carbon foam with microporous structure for high performance symmetric potassium dual-ion capacitor. J. Energy Chem. **43**, 129-138 (2020). https://doi.org/10.1016/j.jechem.2019.08.013

[S25] Y. Luo, D. Huang, C. Liang, P. Wang, K. Han et al., Fe_2VO_4 hierarchical

porous microparticles prepared via a facile surface solvation treatment for high-performance lithium and sodium storage. Small **15**, e1804706 (2019). https://doi.org/10.1002/smll.201804706

[S26] X. Li, J. Xu, L. Mei, Z. Zhang, C. Cui et al., Electrospinning of crystalline MoO_3@C nanofibers for high-rate lithium storage. J. Mater. Chem. A **3**, 3257-3260 (2015). https://doi.org/10.1039/C4TA06121G

[S27] X. Liu, G. Li, D. Zhang, L. Meng, B. Li et al., F doped Li_3VO_4: An advanced anode material with optimized rate capability and durable lifetime. Electrochim. Acta **354**, 136655 (2020). https://doi.org/10.1016/j.electacta.2020.136655

[S28] M.C. Shin, J.H. Kim, S. Nam, Y.J. Oh, H.J. Jin et al., Atomic-distributed coordination state of metal-phenolic compounds enabled low temperature graphitization for high-performance multioriented graphite anode. Small **16**, e2003104 (2020). https://doi.org/10.1002/smll.202003104
